# Effects of Social Skills Training for Adolescents on the Autism Spectrum: a Randomized Controlled Trial of the Polish Adaptation of the PEERS® Intervention via Hybrid and In-Person Delivery

**DOI:** 10.1007/s10803-022-05714-9

**Published:** 2022-08-24

**Authors:** Mateusz Płatos, Kinga Wojaczek, Elizabeth A. Laugeson

**Affiliations:** 1 Faculty of Psychology, University of Warsaw, Stawki 5/7, 01-909 Warsaw, Poland; 2 Association for Social Innovation “Mary and Max”, Marszałkowska 84/92/201, 00-514 Warsaw, Poland; 3 Department of Psychiatry and Biobehavioral Sciences, Semel Institute for Neuroscience and Human Behavior, University of California, 300 UCLA Medical Plaza, 90095-6967 Los Angeles, CA USA

**Keywords:** Autism Spectrum, Social Skills Training, PEERS, Adolescence, Hybrid Delivery

## Abstract

The study examined the efficacy of the Polish adaptation of the PEERS® curriculum for adolescents on the autism spectrum. Twenty-nine adolescents (aged 11–16) were randomized into a Treatment and a Waitlist Control Group. Due to COVID-19-related restrictions, the Treatment Group received part of the intervention online (in hybrid mode). Results showed large effects of PEERS® increasing the teens’ social skills, knowledge about social skills, and the number of get-togethers with peers. Most of the effects were maintained over a six-month follow-up period. There was no impact of the delivery mode on the treatment effects. The study demonstrates the feasibility and efficacy of the Polish adaptation of PEERS® and encourages future research on the online/hybrid delivery of Social Skills Training.

**Supplementary Information:**

The online version contains supplementary material available at 10.1007/s10803-022-05714-9.

Autism spectrum is a common developmental condition characterized by persistent social and communication difficulties, as well as restricted, repetitive patterns of interests, activities, and behavior (World Health Organization [WHO], 2022). There is a growing recognition that people on the autism spectrum also have unique strengths (Pellicano & den Houting, 2022), such as an ability to systemize, good attention to detail, or intrinsic motivation to pursue their interests (Baron-Cohen et al., 2009; Patten Koenig & Hough Williams, 2017), and those strengths should be leveraged in planning support for autistic individuals (Lanou, Hough, & Powell, 2012).

For autistic people, adolescence is often a period of painful mismatch between their abilities and interests and expectations of their peer group (Cresswell et al., 2019). Increasing social demands impede initiating and maintaining friendships that require skills such as holding a two-way conversation, communicating via social media, or appropriate use of humor. It is in peer relationships where recognizing and applying non-explicit social norms is most difficult and social mistakes can quickly lead to a bad reputation, exclusion, and bullying (Maiano et al., 2016; Symes & Humphrey, 2010). Consequently, peer problems among adolescents on the autism spectrum contribute to high rates of depression, social anxiety, and low self-esteem (Hebron et al., 2015; Storch et al., 2012).

Social Skills Training (SST) is a common, psychosocial intervention for adolescents on the autism spectrum. It involves structured teaching of knowledge and skills related to social relationships, usually in a group setting (Ellingsen et al., 2017). SST accumulated a large body of evidence and is considered an evidence-based treatment (Gates et al., 2017; Hume et al., 2021), although concerns have been raised regarding the external validity of the existing studies (Jonsson, Choque Olsson, & Bölte, 2016), as well as cultural adaptation and dissemination of SST (Davenport et al., 2018). Specifically, Davenport and colleagues have found only five studies evaluating the efficacy of SST for autistic individuals (and two for adolescents) that had been culturally adapted, including single-case studies.

Consistent with these findings, there is no evidence-based SST, either adapted or original, for autistic adolescents available in Poland. Nevertheless, about 46% of adolescents and adults on the autism spectrum report using group treatment, including mostly SST (Płatos & Pisula, 2019), but their quality and efficacy are usually unknown. Importantly, teaching social skills that are not ecologically valid can lead to peer rejection and, in some cases, even worsening adolescents’ social functioning (Laugeson et al., 2014).

The Program for the Education and Enrichment of Relational Skills (PEERS®) is a manualized, parent-mediated group social skills training developed at the University of California – Los Angeles in the United States (Laugeson & Frankel, 2010). The program focuses on the skills needed to initiate and maintain peer relationships, such as conversational skills, electronic communication, or resolving conflict. Teaching techniques and materials are adapted to autistic students’ learning styles that encompass visual thinking, good memory, and systemizing. In particular, the curriculum is structured, and each skill is presented in concrete, explicit rules or steps. These rules and steps are first discussed and shown to adolescents in role-play demonstrations. Next, the skills are practiced in behavioral rehearsal exercises and homework assignments involving parent coaching and socialization with peers. Teens’ interests are used as the primary motivation to engage in peer relationships (doing things together and talking about common interests), which is in line with the understanding of friendship by many adolescents on the autism spectrum (Płatos & Pisula, 2021).

The efficacy of the PEERS® for Adolescents program was confirmed in two original RCTs conducted by its founders (Laugeson et al., 2009, 2012), that were then replicated and expanded in several studies (Zheng et al., 2021). According to teen and parent reports, participants of the PEERS® program improved their social skills, knowledge of social rules, and frequency and quality of their get-togethers with friends. The feasibility of cultural adaptation of the intervention was confirmed in RCTs conducted in China (Shum et al., 2019), South Korea (Yoo et al., 2014) and Israel (Rabin et al., 2018). Surprisingly, despite the popularity of the PEERS® program in Europe (UCLA Semel Institute for Neuroscience and Human Behavior, n.d.), to date, only one RCT on its efficacy has been published in a European country (the Netherlands; Idris et al., 2022).

The current trial was designed to examine ecological validity and efficacy of the Polish version of the PEERS curriculum. The main rationale was to address a need for culturally adapted, highly acceptable, evidence-based SST for adolescents on the autism spectrum. However, the study was conducted in the uncertain time of the COVID-19 pandemic. By natural experiment, a lockdown imposed by the Polish government forced the authors to lead experimental groups in hybrid mode (a part of the classes were held online), while control groups received their delayed intervention entirely in-person. Besides the main focus of the study, those circumstances allowed for examination of the potential impact of the treatment delivery on the therapeutic effects.

There is emerging evidence on the efficacy of psychosocial interventions for autistic people delivered using telehealth tools. In a recent systematic review, Ellison et al., (2021) identified four telehealth intervention studies directly engaging autistic children or adolescents. These interventions showed effects in, for example, reducing children’s anxiety and sleep problems but provided no comparison with in-person services. Establishing an evidence base for telehealth social skills training can be useful not only in the context of the future pandemic threat but can also help decrease costs of services and overcome geographical distance, which have been identified as the main barriers to services by autistic individuals and their families (Płatos & Pisula, 2019).

The goals of the study were to (a) examine the efficacy and ecological validity of the Polish adaptation of the PEERS® for Adolescents curriculum, (b) evaluate the maintenance of the intervention effects over time, and (c) explore the potential impact of the treatment delivery (hybrid vs. in-person). We hypothesized that as a result of the treatment, adolescents on the autism spectrum would increase (a) social and communication skills, in particular, those related to peer relationships, (b) knowledge about social norms and expectations related to peer relationships, and (c) the number of get-togethers with peers, but would decrease (d) their level of conflict with peers during get-togethers. We expected that these effects would maintain over a six-month period. The analysis of the impact of the treatment delivery was unplanned and exploratory in nature, but based on the previous literature (Ellison et al., 2021; Estabillo et al., 2022), we foresaw comparable effects of the intervention in hybrid and in-person delivery modes.

## Methods

### Recruitment and participants

Participants were recruited through advertisements in local therapeutic centers, schools, and social media, as well as through referrals from psychologists and psychiatrists. Written, informed consent to take part in the study was obtained from both parents and teens. Inclusion criteria for adolescents included: (a) age of 11–18 years; (b) clinical diagnosis of Pervasive Developmental Disorder (including Asperger Syndrome, childhood autism, and atypical autism), according to ICD-10 (WHO, 1992), confirmed by a licensed child and adolescent psychiatrist; (c) absence of co-existing intellectual disability (IQ > 70); (d) absence of a major, concurrent psychiatric disorder (e.g., schizophrenia, bipolar disorder); (e) absence of oppositional/aggressive behavior (out of family context); (f) having difficulties in developing and/or maintaining peer relations; (g) self-motivation to participate in the treatment; (h) availability of a caregiver that is willing to regularly participate in parent sessions and support the participant throughout the program; and (i) parental and teen consent to take part in all the assessments and to be recorded during the treatment sessions.

In line with guidelines from the PEERS® for Adolescents manual (Laugeson & Frankel, 2010), parents of candidates were first screened via phone interview. If the initial criteria were met, a parent and a teen were invited to an intake interview with a psychologist who evaluated the participant’s motivation, risk of challenging behaviors, and mental health status. The parent’s availability and readiness for the role of a social coach were also assessed during the interview. The third phase of the screening process involved standardized assessments. The autism spectrum diagnosis was confirmed by the administration of the Autism Diagnostic Observation Schedule, Second Edition (ADOS-2; module 3 or 4; Chojnicka and Pisula, 2017) provided by certified diagnosticians. The cognitive and adaptive functioning was assessed using the Abbreviated Battery of the Stanford-Binet Intelligence Scales, Fifth Edition (Sajewicz-Radtke et al., 2017), and the Adaptive Behavior Assessment System, Third Edition (ABAS-3; Otrębski et al., 2019), respectively.

**Fig. 1:**
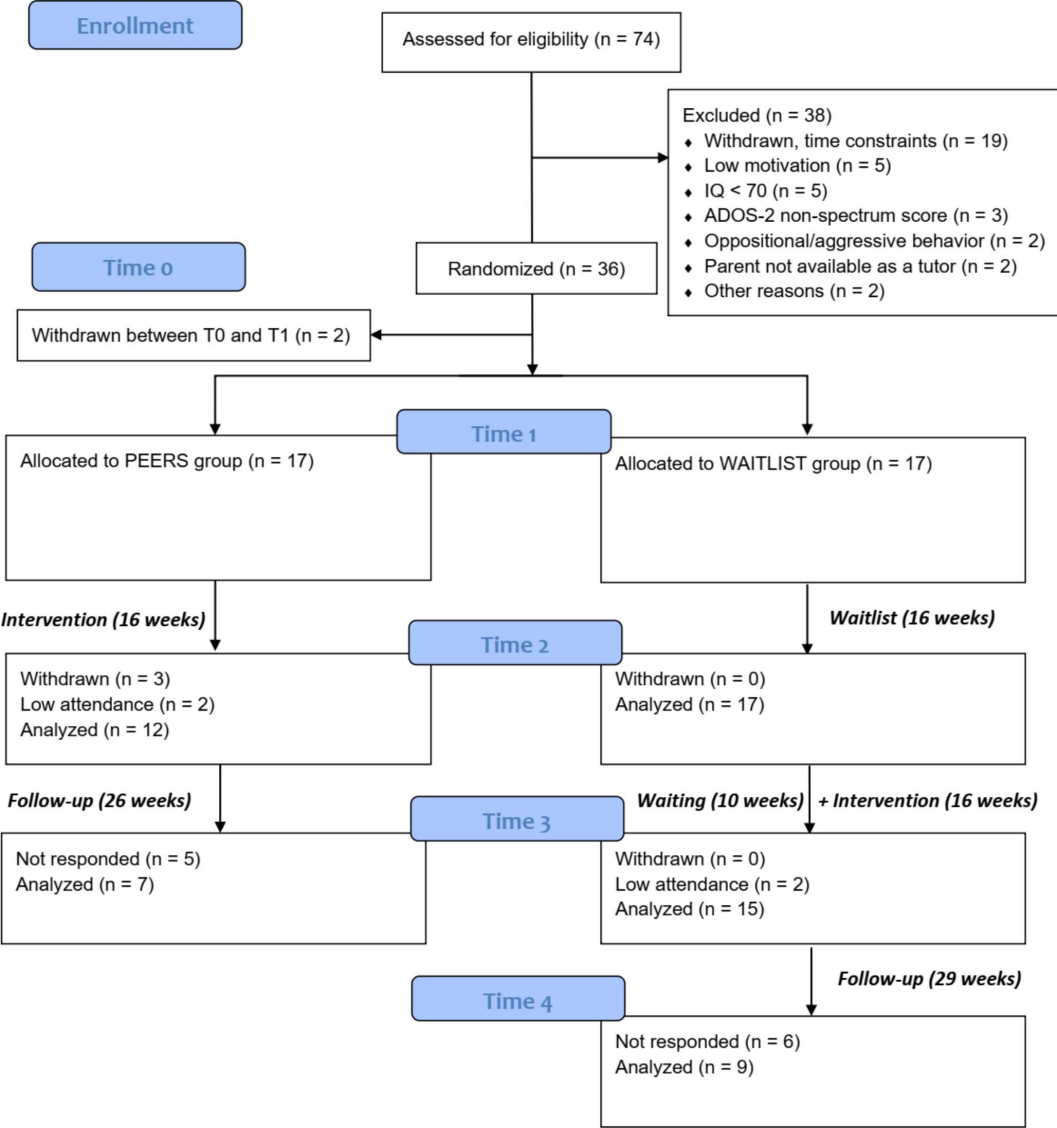
CONSORT flow diagram

As shown in Fig. 1, out of 74 adolescents assessed for eligibility, 36 were accepted to the study and 38 were excluded, mainly because of time/scheduling constraints (*n* = 19), low motivation (*n* = 5), or IQ below 70 (*n* = 5). Two participants withdrew after randomization during the COVID-related waiting period (see the section below). During the intervention period, three participants withdrew from the study (8.8%), all allocated to the treatment group. Additional two participants from the treatment group were excluded from the analyses due to low attendance (< 13 sessions).

The final sample consisted of 29 adolescents aged between 11.8 and 16.1 years (62.1% males; all Caucasian/White) before the intervention (T1). Participants attended mainstreamed (68.9%), integrated (24.1%), and in two cases special education (6.9%) classrooms. The majority had a diagnosis of Asperger Syndrome (86.2%) and 34.5% had at least one co-existing developmental or psychiatric disorder (ADD/ADHD: *n* = 6; depression: *n* = 2; OCD/other anxiety disorders: *n* = 2). Reporting parents included 25 mothers (including one adoptive parent) and four fathers, mostly with tertiary education (82.8%). Detailed demographic information on the Treatment and Waitlist Control groups are presented in Table 1.

**Table 1:** Characteristics of participants at baseline (T1)

Variables	Treatment Group (*n* = 12)	Waitlist Control Group (*n* = 17)	
	*M (SD)*	*M (SD)*	*p*
Age (years)	14.6 (1.0)	14.3 (1.2)	ns
Gender (% male)	66.7	58.8	ns
ASD diagnosis (%)			ns
Asperger Syndome	75.0	94.1	
Childhood autism	25.0	5.9	
Comorbid psychiatric disorders (%)	33.3	35.3	ns
On medication (%)	41.7	23.5	ns
Parental age (years)	46.8 (5.7)	47.3 (4.4)	ns
Parental education (% tertiary)	100.0	70.6	ns
*Baseline Measures*			
ADOS-2 Comparison Score	6.67 (2.00)	5.93 (1.53)	ns
Stanford-Binet 5 Matrices	10.22 (1.56)	11.58 (2.31)	ns
Stanford-Binet 5 Vocabulary	11.00 (2.06)	12.58 (3.37)	ns
Stanford-Binet 5 Total IQ	104.67 (7.31)	113.50 (14.46)	ns
ABAS-3 Communication	52.90 (8.19)	56.82 (6.13)	ns
ABAS-3 Social	46.70 (8.82)	51.35 (9.01)	ns
ABAS-3 Self-direction	51.90 (12.64)	49.47 (10.00)	ns
*Adolescent Report Outcome Measures*			
TASSK-R	16.33 (1.88)	15.82 (2.22)	ns
QSQA-R Total get-togethers	1.27 (1.95)	2.31 (2.75)	ns
QSQA-R Conflict	4.10 (2.56)	5.50 (5.11)	ns
*Parent Report Outcome Measures*			
QSQP-R Total get-togethers	1.33 (1.87)	2.17 (2.53)	ns
QSQP-R Conflict	12.11 (6.70)	9.69 (3.77)	ns
SRS-2 Total	115.50 (28.77)	95.24 (21.82)	0.040
SRS-2 SCI	95.25 (23.25)	77.41 (17.53)	0.026
SRS-2 RRB	20.25 (6.27)	17.82 (5.63)	ns
ASRS Total	114.17 (29.00)	102.18 (25.07)	ns
ASRS Social/Communication	41.08 (8.30)	35.47 (10.64)	ns
ASRS Unusual Behaviors	39.08 (15.29)	33.65 (11.37)	ns
ASRS Peer Socialization	23.33 (5.03)	19.82 (4.69)	ns

Note. ns = statistically non-significant. TASSK-R = Test of Adolescent Social Skills Knowledge (Revised); QSQA/P-R = Quality of Socialization Questionnaire (Revised); SRS-2 = Social Responsiveness Scale-2; ASRS = Autism Spectrum Rating Scale

### Study design and procedures

After the screening process, participants eligible for the study were randomly allocated to the Treatment Group or Waitlist Control Group. The randomization sequence was generated using Sealed Envelope online software (Sealed Envelope Ltd, 2021) with a 1:1 allocation using random block sizes of 2 or 4, stratified by age group (11–13 and 14–16 years of age). The procedure was conducted by the independent allocator, who knew only the ID number, age group, and sex of the participants. The last variable was used to avoid a situation when there was only one female or one male in a group. The group allocation was concealed to participants, their parents, and the staff administering standardized tests until participants completed the baseline measures (T0).

Although the Treatment Group was to receive the treatment immediately after the baseline measurement (March 2020), the COVID-19-related restrictions imposed by the Polish government forced the researchers to postpone the intervention by six months. Therefore, the baseline measurement was repeated (T1) to ensure that the groups remained balanced in terms of demographic and outcome measures (see “Results”). Both groups were assessed again immediately after the Treatment Group received the treatment (T2) and then after the Waitlist Group received the treatment (T3). The time between T2 and T3 included an additional 10-week waiting period due to further COVID-19-related restrictions (10-weeks + 16-weeks = 26-weeks). Finally, the Waitlist Control Group was assessed for the maintenance of the results 29-weeks after the intervention (T4). The study design is shown in Fig. 1.

All the adolescent groups were led by a PEERS® Certified Provider (first author), who previously served as an intern at UCLA, was trained by the program developer, and assisted in facilitating PEERS® treatment groups at UCLA. The group leader was aided by two clinical assistants who were psychology students and were trained and supervised by the first author. The parent groups were led by a psychologist who participated in the program adaptation and was trained by the first author during the program’s pilot.

### The PEERS® program and its adaptation

PEERS® for Adolescents is a parent-assisted social skills training that uses cognitive-behavioral techniques to teach ecologically valid social skills to adolescents on the autism spectrum (Laugeson & Frankel, 2011). The focus of the program is to help teens develop and maintain peer relationships. The curriculum includes conversational skills, electronic communication, appropriate use of humor, peer entry and exiting, good sportsmanship, organizing get-togethers, handling arguments, changing a reputation, and handling different types of bullying (see Table S1 for the full curriculum). In each of the fourteen 90-minutes sessions, a therapist presents relevant skills through direct instruction, Socratic questioning, and role-play demonstrations. Participants practice skills in behavioral rehearsal exercises and receive feedback on their performance from the treatment team. They are also assigned weekly homework to complete between the sessions, involving practicing skills with their parents and socialization with peers (e.g., to have a phone conversation with another group member or organize a get-together with a friend). Parents (or other adult family members, called “’social coaches” in the program) participate in the parallel sessions and learn how to facilitate the skills acquisition of their teens. Both homework assignments and parent assistance foster generalization and maintenance of the learned skills.

To ensure ecological and cultural validity of the Polish version of the program, two kinds of revisions were required: (a) adaptation of culturally sensitive content of the manual and (b) updating session content to reflect changes in the social context since the original conception of the program. Previous adaptions of the PEERS® program showed that only minor changes were necessary due to cultural differences, such as changing jokes used by adolescents or crowds they might belong to (Rabin et al., 2018; Shum et al., 2019; Yoo et al., 2014). In contrast, phenomena such as social media, online gaming, and most recently the COVID-19 pandemic have radically changed the way young people interact with each other and maintain relationships. Therefore, a multisource approach was used to create the most current and ecologically valid curriculum. The original manual (Laugeson & Frankel, 2010) remained the primary source of the adaptation but was supplemented by the following materials: (a) the updated PEERS® curriculum for School-Based Professionals (Laugeson, 2013), (b) the PEERS® for Young Adults manual (Laugeson, 2017), (c) materials from a PEERS® Certified Training Seminar (2018), (d) the telehealth version of the PEERS® curriculum, shared by its author (Laugeson, 2020). These materials included updated session content, such as new rules and steps that were incorporated into the adapted version of the Polish PEERS® curriculum.

The adaptation procedure involved (a) translation of each of the PEERS® manual chapters by the first author, (b) incorporating updated contents from other sources, (c) 3-hour consensus meetings on each session with the second author to review the translation and propose relevant changes, (d) consulting about potentially sensitive elements of the program with other specialists, neurotypical young people and autistic self-advocates as needed, (e) translation and adaptation of all the auxiliary materials (homework worksheets, intake forms etc.), (f) preparing a Polish version of over 100 short role-play videos that present social skills (and common mistakes) taught in the program. Major changes to the original manual included expanding the program to 16-sessions, discontinuing a token economy used to reinforce participants’ activity (as this was perceived by participants as too competitive and stressful), as well as referring to the program as a “social skills workshop” instead of “social skills training” because the latter name had negative connotations for some of the consulted self-advocates. A full review of the program modifications is presented in Table S1.

Further changes had to be made as a direct consequence of the COVID-19 pandemic. These changes affected mostly the Treatment Group (during a period between T1 and T2 when the second and third wave of the coronavirus spread over Poland) allowing for a preliminary assessment of their impact on the intervention outcomes (see “Results”). Namely, last 7 out of 16 sessions in the Treatment Group were taught using synchronous online tools, and the rest were held in person. All the participants received the same number of online and in-person sessions. No modifications were made to the didactic content of the sessions, but there were minor changes in the homework assignments. In particular, participants could organize virtual get-togethers with peers and they could invite also other PEERS® participants, which is not recommended in the original PEERS® manual (Laugeson & Frankel, 2010). The Waitlist Control Group received the treatment in a traditional in-person setting, although participants who were sick or on quarantine could still participate online (on average, one session out of 16 was attended online).

### Outcome Measures

*Test of Adolescent Social Skills Knowledge—Revised (TASSK-R*: Laugeson et al., 2012).

The TASSK-R is a self-report, criterion-referenced measure that assesses teens’ knowledge about social skills taught throughout the program. It consists of 26 sentence stems in which adolescents are asked to choose from two possible answers. A higher score indicates more knowledge about social skills related to the treatment. The TASSK-R is a revised version of the original measure developed specifically to test the efficacy of the PEERS® curriculum (Laugeson & Frankel, 2010) and proved to be sensitive to change in previous studies (Laugeson et al., 2012; Shum et al., 2019; Yoo et al., 2014). In the current sample, at T1, the measure showed very low internal consistency, similar to the previous studies (Laugeson et al., 2009; Schohl et al., 2014; Shum et al., 2019). This probably happened because participants did not know the answers and responded randomly, thereby decreasing covariances between the items. In contrast, after participants completed the treatment, the alpha coefficients increased to an acceptable level (T3 = 0.77).

*Quality of Socialization Questionnaire-Revised (QSQ-R*: Laugeson 2018*)*.

The QSQ-R assesses the frequency and quality of the teen’s get-together with friends, as reported by adolescents (QSQA-R) and parents (QSQP-R). Two items ask the individual to report the number of hosted and invited get-togethers with peers that the teen held over the last month. The answers to these two questions were summed up to form a single variable (total number of get-togethers). To check the reliability of the report, teens and parents were also asked to provide a list of names of the friends the teen met with. As a part of the intervention took place during pandemic-related restrictions in movement and social gatherings, participants were allowed to report both in-person and online get-togethers (e.g., using video conference). Additionally, the measure includes a 12-item questionnaire that assesses the level of conflict during the most recent get-together with a peer, with answers provided on a 4-point Likert scale, ranging from “Not at all true” to “Very much true.“ In the current sample, the scale showed acceptable internal consistency in adolescent (Cronbach’s α = 0.70) and parent (α = 0.73) versions of the measure.

*Social Responsiveness Scale-2 (SRS-2;* Constantino & Gruber 2012*)*.

The SRS-2, School-Age Form, is a 65-item rating scale used to measure autism symptoms and severity. It is intended for parents of children between 4 and 18 years and takes about 15 min to complete. Each sentence is rated on a 4-point Likert scale, ranging from “Not at all true” to “Almost always true.“ A higher score reflects more autism symptoms. The SRS-2 consists of the Total score, two DSM-5 Compatible Subscales (Social Communication and Interaction, Restricted Interests and Repetitive Behavior), and five Treatment Subscales (Social Awareness, Social Cognition, Social Communication, Social Motivation, Restricted Interests and Repetitive Behavior). The questionnaire was translated (MP) and then back-translated by an independent translator for the purpose of the present study. All discrepancies were resolved with the authors of the original measure. The SRS-2 was used in the previous evaluations of the PEERS® curriculum and proved to be sensitive to the treatment effects (Corona et al., 2019; Rabin et al., 2018). The reliability of the SRS-2 (Total Score) in the current sample was high (Cronbach’s α = 0.93).

*Autism Spectrum Rating Scale (ASRS;* Goldstein & Naglieri 2010*)*.

The ASRS (for ages 6–18) is a 71-item rating scale intended to measure behaviors associated with autism spectrum. In the current study, it was completed by parents who rated each sentence on a 5-point Likert scale ranging from “Never” to “Very often.“ A higher score indicates more autism symptoms. The ASRS consists of the Total Score and several subscales, including nine Treatment Scales. In the current study, the Peer Socialization Subscale was hypothesized to be particularly sensitive to change following the PEERS® Curriculum. The Polish adaptation of the questionnaire was prepared by Wrocławska-Warchala & Wujcik (2016) on a sample of about 1500 parents and yielded high internal consistency (Cronbach’s α > 0.80). Similar coefficients were attained in the present study (Total Score: α = 0.92; Peer Socialization: α = 0.72).

### Ecological validity

To assess the program’s ecological validity, after the treatment, adolescents and parents filled out an evaluation survey prepared for the study. The survey included seven close-ended questions, rated on a 7-point Likert scale, concerning participants’ satisfaction with various elements of the program, time burden, overall satisfaction, and willingness to recommend the program to other teens on the autism spectrum or their parents (see Table S2 in Appendix for details). The survey also included two open-ended questions regarding positive aspects of the program and things that could be improved or changed.

### Treatment fidelity

All the sessions were videotaped and the quality of the treatment implementation was monitored by the first author (PEERS® Certified Provider). Participants’ adherence to treatment was controlled by recording teens’ and social coaches’ attendance and rates of completion of homework assignments. In both TG and WCG, participants attended a median number of 15 out of 16 sessions (93.8%), including those with low attendance. Waitlist control groups were conducted fully in person, but during illness and quarantine, participants could still participate online. Teens used this opportunity, on average, once during a program. The homework completion rate was high, regardless of the treatment delivery mode. For example, for six consecutive weeks teens were asked to call another participant, exchange information, and find common interests. In TG and WCG, completion rates of this homework were 95.2% and 98.0%, respectively.

### Data analysis

Two-way mixed analyses of variance (ANOVAs) were used to ascertain the treatment effect. When a statistically significant interaction effect (Group x Time) was found, it was followed by simple effects analyses. The level of significance for the Box’s M Test was set to 0.001, due to the sensitivity of the assumption of homogeneity of variance-covariance matrices (Verma, 2015). To account for multiple comparisons, the Benjamini-Hochberg procedure was applied in all the analyses, with the False Discovery Rate (FDR) set at 0.10 (Benjamini & Hochberg, 1995). Statistical analyses were conducted using IBM SPSS 27 package and qualitative analyses were conducted using ATLAS.ti 9 software.

## Results

### Preliminary analyses

All the variables were tested for extreme values. Only in QPQA-R and QPQP-R 12 and 14 potential outliers across all time points (T0-T4) were found (3.7% and 4.2% of all data points), respectively. As adolescents and parents were asked to provide a list of names of peers that adolescents met with, the lists were used to validate the numerical data. Four results from the QPQA-R and one result from the QPQP-R were assessed as genuine outliers and remained in the analyses. The rest of the data points were corrected using the winsorization procedure (Wilcox, 2005).

There were very few missing values on single items (< 3% of responses) except for three items of the teen-reported conflict scale at T1 (QSQA-R; 7.1% in two items, 10.1% in one item). All the missing values were missing completely at random, as shown by Little’s test (Little, 1988), and computed using the Expectation-Maximization procedure (Dempster et al., 1977).

The differences between participants who were excluded or who withdrew from the study and those who completed it were ascertained using an independent-samples t-test or Mann-Whitney U test (for variables with non-normal distribution). There were no statistically significant differences between those groups in any demographic variables. Similarly, there were no differences in ADOS-2 and IQ scores or outcome variables at baseline (T0).

The comparability of the Treatment Group and Waitlist Control Group was examined both at the first (T0) and repeated baseline (T1). At T0, chi-square tests for association between the group allocation and participants’ gender, autism spectrum diagnosis, comorbid psychiatric disorders, the use of the psychotropic medication, and parental education yielded no significant results (*p* > .05). T-tests for between-group differences in participants’ age, parental age, ADOS-2 Comparison Score, intelligence scores (verbal, performance, and total), ABAS-3 subscales, and all the outcome measures were also not significant. These results were replicated at T1, except for the SRS-2, in which some differences reached significance (see Table 1). Therefore, it can be concluded that the waiting period during the pandemic-related lockdown did not affect group comparability.

Parents were asked about other treatments their teen received between T1 and T2. About one-third in both groups (Treatment Group = 33.3%; Waitlist Control Group = 35.3%) reported concurrent treatments, mostly psychotherapy, and the between-group difference was not statistically significant (*χ*^*2*^(1) = 0.012; *p* = .913). One parent in the Treatment Group and two in the Waitlist Control Group described the concurrent treatment as a social skills training. The average time of all received treatments was estimated as one hour per week in the Treatment Group and 0.3 h per week in the Waitlist Control Group and was not statistically significantly different (Mann-Whitney *U* = 106.00; *z* = 0.209; *p* = .834).

### Treatment efficacy

**Fig. 2:**
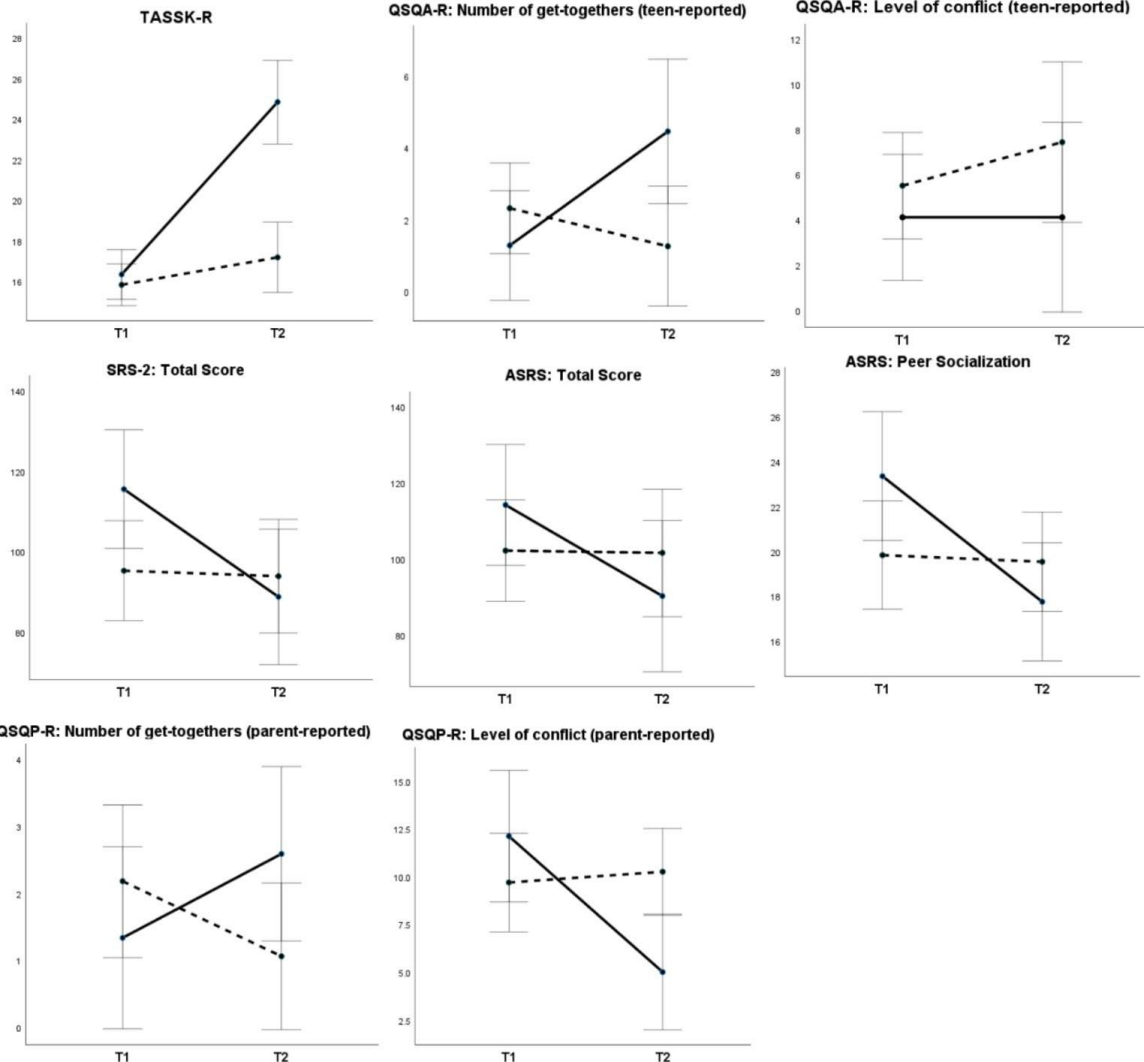
Means and standard errors for outcome variables in the Treatment (solid line) and Waitlist Control Group (dotted line) at Pre-test (T1) and Post-test (T2). TASSK-R = Test of Adolescent Social Skills Knowledge (Revised); QSQA-R = Quality of Socialization Questionnaire—Adolescent (Revised); SRS-2 = Social Responsiveness Scale-2; ASRS = Autism Spectrum Rating Scale; QSQP-R = Quality of Socialization Questionnaire—Parent (Revised)

To test the immediate effects of the PEERS® curriculum, two-way mixed analyses of variance were conducted with Group as a between-subjects factor and Time as a within-subjects factor. Statistically significant interactions (Group x Time) were found for adolescents’ self-reported knowledge about social skills (TASSK-R), the self- and parent-reported number of get-togethers with peers and the parent-reported level of conflict (QSQA-R and QSQP-R), a parent-reported severity of autism symptoms (SRS-2 Total Score and ASRS Total Score), and a parent-reported quality of peer socialization (ASRS Treatment Subscale), with large effect sizes (η^2^ > 0.14). Detailed results of these analyses are presented in Table 2; Fig. 2.

**Table 2:** Means, standard deviations, and mixed ANOVAs results for treatment and waitlist control groups

Outcome variables	Treatment (*n* = 12)		Control (*n* = 17)				
	Pre (T1)	Post (T2)	Pre (T1)	Post (T2)			
	*M (SD)*	*M (SD)*	*M (SD)*	*M (SD)*	Time x Group	*p*	*η*_*p*_^*2*^
*Teen report*							
TASSK-R	16.33 (1.88)	24.83 (4.15)	15.82 (2.22)	17.18 (2.94)	F(1, 27) = 31.61	< 0.001*	0.539
QSQA-R (get-togethers)	1.27 (1.95)	4.45 (4.59)	2.31 (2.75)	1.25 (1.87)	F(1, 25) = 10.24	0.004*	0.291
QSQA-R (conflict)	4.10 (2.56)	4.10 (3.28)	5.50 (5.11)	7.43 (7.88)	F(1, 22) = 0.426	0.521	0.019
*Parent report*							
SRS-2 Total Score	115.50 (28.77)	88.75 (36.88)	95.24 (21.82)	93.88 (20.59)	F(1, 27) = 9.48	0.005*	0.260
ASRS Total Score	114.17 (29.00)	90.25 (41.03)	102.18 (25.07)	101.59 (27.27)	F(1, 27) = 9.40	0.005*	0.258
ASRS Peer Socialization	23.33 (5.03)	17.75 (4.71)	19.82 (4.69)	19.53 (4.24)	F(1, 27) = 7.20	0.012*	0.210
QSQP-R (get-togethers)	1.33 (1.87)	2.58 (2.81)	2.18 (2.53)	1.06 (1.64)	F(1, 27) = 6.58	0.016*	0.196
QSQP-R (conflict)	12.11 (6.70)	5.00 (4.44)	9.69 (3.77)	10.25 (4.36)	F(1, 23) = 8.33	0.009*	0.227

Note. TASSK-R = Test of Adolescent Social Skills Knowledge (Revised); QSQA-R = Quality of Socialization Questionnaire—Adolescent (Revised); SRS-2 = Social Responsiveness Scale-2; ASRS = Autism Spectrum Rating Scale; QSQA-R = Quality of Socialization Questionnaire—Adolescent (Revised)

* Statistically significant under Benjamini-Hochberg procedure (FDR = 0.10)

Post-hoc simple effects analyses were conducted using univariate, within-subjects ANOVAs. In the Treatment Group, adolescents’ knowledge about social skills significantly increased after the intervention (*F* (1, 11) = 48.41; *p* < .001; partial η^2^ = 0.815), while in the Waitlist Control Group a change remained at a trend level (*F* (1, 16) = 4.53; *p* = .049; partial η^2^ = 0.221). Moreover, the teen-reported number of get-togethers with peers increased in the Treatment Group (*F* (1, 10) = 6.06; *p* = .034; partial η^2^ = 0.378) but decreased in the Waitlist Control Group, although the latter effect was not statistically significant (*F* (1, 15) = 2.63; *p* = .126; partial η^2^ = 0.149). Analogous pattern of results was obtained in the parent-reported number of get-togethers, but time effects did not reach significance (increase in TG: *F* (1, 11) = 4.28; *p* < .063; partial η^2^ = 0.280; decrease in WCG: *F* (1, 16) = 4.28; *p* < .064; partial η^2^ = 0.198). Furthermore, the parent-reported level of conflict during teens’ get-togethers statistically significantly decreased in TG (*F* (1, 8) = 6.91; *p* < .030; partial η^2^ = 0.463), with no change in WCG (*F* (1, 15) = 0.185; *p* < .673; partial η^2^ = 0.012).

Regarding autism-related difficulties, TG showed a statistically significant decrease in SRS-2 Total Score (*F* (1, 11) = 9.46; *p* < .011; partial η^2^ = 0.462), while WCG did not (*F* (1, 16) = 0.17; *p* < .686; partial η^2^ = 0.010). Similarly, TG showed a statistically significant decrease in ASRS Total Score (*F* (1, 11) = 8.43; *p* < .014; partial η^2^ = 0.434), while WCG did not (*F* (1, 16) = 0.05; *p* < .833; partial η^2^ = 0.003). Lastly, the level of autism-related difficulties in peer relations significantly decreased in TG (*F* (1, 11) = 8.42; *p* < .014; partial η^2^ = 0.433), but remained stable in WCG (*F* (1, 16) = 0.09; *p* < .763; partial η^2^ = 0.006). All the statistically significant results (*p* < .05) remained significant after adjustment for multiple comparisons using Benjamini-Hochberg procedure.

### Ecological validity

Both adolescents and their parents rated all the program components as helpful (see Table S2 in Supplementary Materials). Doing homework received the lowest score among adolescents but still above the middle of the scale (4.4 on a 7-point scale). The program was rated as relatively time-consuming but not too burdening by teens (3.3 on a scale from 1 = “little burden” to 7 = “too much burden”) and parents (3.6). Both parties reported that by participating in the PEERS® program, teens learned to establish and maintain friendships better. All the parents and 70.4% of adolescents (n = 19) would recommend the program to others (scores 6 or 7 on a scale from 1 = “definitely not” to 7 = “definitely yes”), with two adolescents that would “definitely not” recommend it. There were no significant differences between the TG, which received the program in hybrid mode, and the WCG, which received the program fully in person. However, there were some differences between teens and parents, with the parental ratings of program general helpfulness being higher than the teens’ ratings (Wilcoxon signed-rank test: *T* = 24.0, *z* = -2.31, *p* = .021). Detailed results can be found in Table S2, in Supplementary Materials.

In open-ended questions, most participants (*n* = 12) appreciated didactic methods used in the program as well-tailored to their needs, for example:

*[I liked] practical exercises and those funny role-plays and videos. The program was suitable for people of my age. [I liked] sharing experiences with other group members.*

*[I liked] “solid” and easy-to-learn rules.*

Other participants underscored the openness and positive attitude of the group leaders and pleasant interactions with other students. Similarly, parents valued sharing experiences with other parents (*n* = 6) and homework reviews (*n* = 5), as well as being provided with concrete rules and steps (*n* = 5) and role-play videos (*n* = 7) to view and practice with their teens.

Parents’ suggestions for the program improvement included adding more time to the meetings or prolonging the program (*n* = 7). However, some parents (*n* = 4) indicated that sometimes group members spent too much time talking about their experiences (not directly related to the program) and avoiding this could save more time for going through the didactic material.

### Maintenance of the treatment effects

Both TG and WCG were tested about six-months after concluding the treatment (T3 and T4, respectively) to ascertain the maintenance of its effects. The response rate was 58.3% and 60.0%, respectively. To avoid inflating a type II error (lack of statistical power to detect differences), whole-group analyses were conducted using within-subjects ANOVAs between pre-post (P1), post-test (P2), and follow-up (P3) with pairwise comparisons. Consistent with the above results, all the main effects of time were large (η^2^ = 0.219-0.701) and significant for all the variables except for the level of conflict (teen-reported). For the rest of the variables, the differences between P1 and P2 (intervention period) were significant (*ps* < 0.019). Differences between P2 and P3 (follow-up period) were insignificant (*ps* > 0.05) except for the number of get-togethers (teen-reported) that decreased. Lastly, most of the differences between P1 and P3 remained significant (*ps* < 0.15) except for the number of get-togethers (teen- and parent-reported) and ASRS Peer Socialization subscale (*p* = .64).

**Table 3:** Maintenance of treatment effects (whole-group analyses; *n* = 16)

	Pre (P1)	Post (P2)	Follow-up (P3)	P2-P1		P3-P2		P3-P1	
	*M (SD)*	*M (SD)*	*M (SD)*	*M*_*d*_ *(SE)*	*p*	*M*_*d*_ *(SE)*	*p*	*M*_*d*_ *(SE)*	*p*
*Teen report*									
TASSK-R	16.56 (3.08)	23.19 (4.92)	22.50 (4.83)	-6.63 (1.00)	< 0.001*	-0.69 (0.50)	0.187	5.94 (1.01)	< 0.001*
QSQA-R (get-togethers)	1.31 (1.85)	5.50 (3.63)	2.37 (3.42)	4.19 (0.97)	< 0.001*	-3.12 (1.34)	0.034*	1.06 (0.76)	0.180
QSQA-R (conflict)	6.29 (4.25)	5.21 (4.06)	5.36 (3.30)	-1.07 (1.23)	0.397	0.14 (1.14)	0.903	− 0.093 (1.34)	0.501
*Parent report*									
SRS-2 Total Score	100.13 (29.53)	81.31 (26.68)	84.19 (30.91)	-18.81 (7.18)	0.019*	2.88 (4.69)	0.549	-15.94 (5.08)	0.007*
ASRS Total Score	99.19 (28.40)	73.06 (26.90)	78.25 (28.73)	-26.12 (6.10)	< 0.001*	5.19 (4.57)	0.274	− 0.20.94 (5.79)	0.003*
ASRS Peer Socialization	21.19 (5.54)	16.25 (5.87)	18.19 (6.57)	-4.94 (1.53)	0.006*	1.94 (1.03)	0.080	-3.00 (1.50)	0.064
QSQP-R (get-togethers)	1.19 (1.83)	3.13 (2.42)	2.50 (3.20)	-1.93 (0.57)	0.004*	-0.63 (0.72)	0.398	1.31 (0.74)	0.096
QSQP-R (conflict)	9.71 (4.68)	3.71 (3.24)	5.71 (4.73)	-6.00 (1.56)	0.002*	2.00 (0.93)	0.050*	-4.00 (1.43)	0.015*

Note. TASSK-R = Test of Adolescent Social Skills Knowledge (Revised); QSQA-R = Quality of Socialization Questionnaire—Adolescent (Revised); SRS-2 = Social Responsiveness Scale-2; ASRS = Autism Spectrum Rating Scale; QSQP-R = Quality of Socialization Questionnaire—Parent (Revised)

* Statistically significant under Benjamini-Hochberg procedure (FDR = 0.10)

**Fig. 3:**
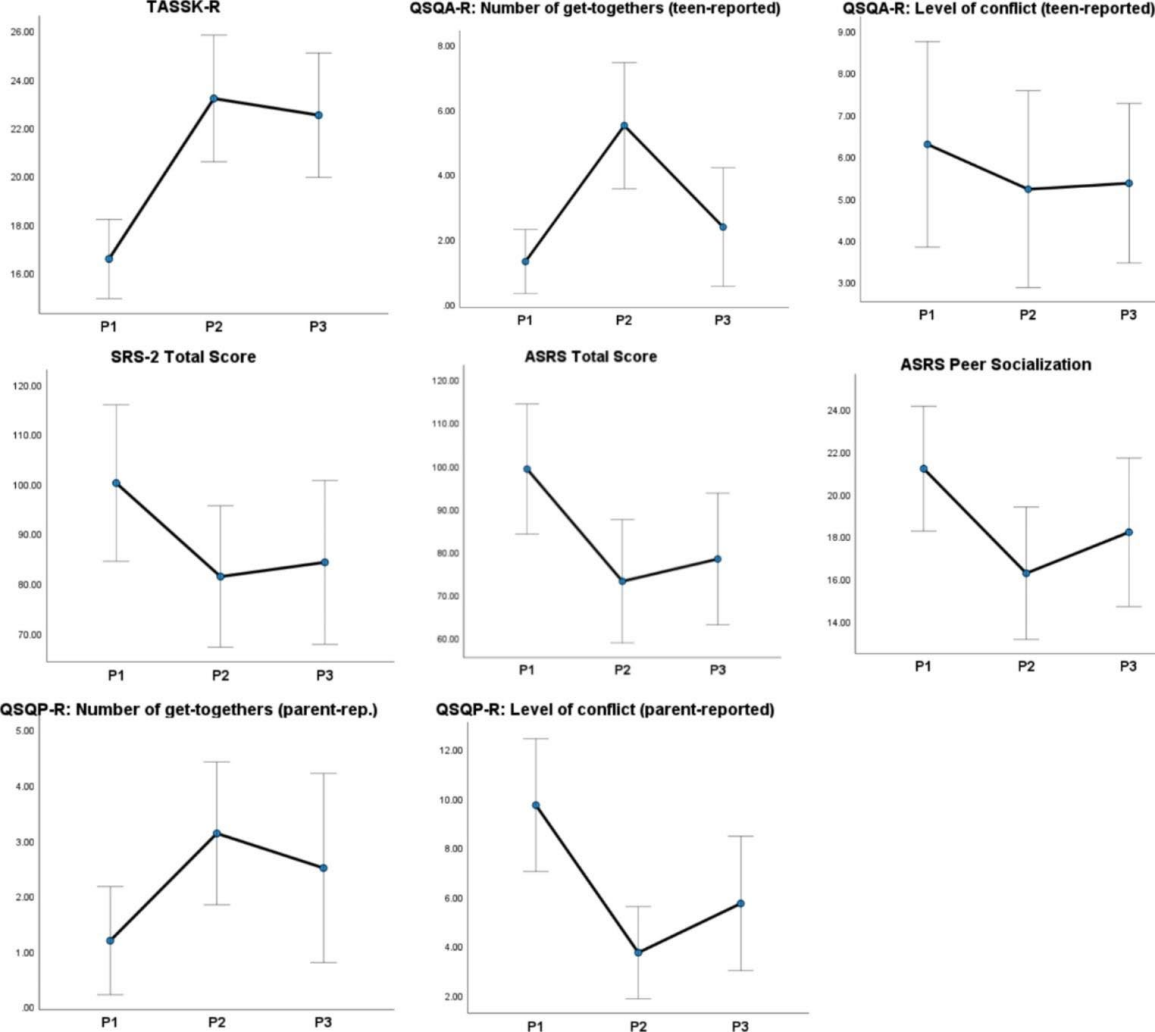
Means and standard errors for outcome variables at Pre-test (P1), Post-test (P2), and Follow-up (P3), Treatment Group and Waitlist Control Group combined. TASSK-R = Test of Adolescent Social Skills Knowledge (Revised); QSQA-R = Quality of Socialization Questionnaire—Adolescent (Revised); SRS-2 = Social Responsiveness Scale-2; ASRS = Autism Spectrum Rating Scale; QSQP-R = Quality of Socialization Questionnaire—Parent (Revised)

### Impact of treatment modality

To test for the potential impact of treatment modality, we compared the efficacy of TG (conducted in hybrid mode) and WCG (conducted in person), using two-way mixed analyses of variance with Group as a between-subjects factor and Time as a within-subjects factor (intervention period for each Group: T1-T2 in TG, T2-T3 in WCG). There were no statistically significant interaction effects, reflecting similar rates of improvement regardless of the mode of treatment delivery. All outcome variables showed no main effects of Group but large main effects of Time, in favor of treatment (range of η^2^ = 0.382-0.819). The exception was a teen-reported level of conflict that showed a main effect of Group but not Time, consistent with previous results.

## Discussion

The main aim of this randomized controlled trial was to examine the efficacy and ecological validity of the Polish adaptation of the PEERS® for Adolescents program. The findings indicate significant improvement in the teens’ social skills, knowledge about social skills, and the number of get-togethers with peers, as reported by adolescents and their parents. Most of the effects were maintained over a six-month period. In addition, the study has also proved that the Polish adaptation of the PEERS® intervention is ecologically valid, as it was both well-accepted and deemed feasible by adolescents and their parents. Lastly, the study explored the potential impact of the treatment delivery during the COVID-19 pandemic, preliminarily showing that the hybrid delivery of treatment is not inferior to the traditional in-person delivery.

The study showed that the Treatment Group was superior to the Waitlist Control Group in most outcome variables. Notably, gains in the teen-reported knowledge of social skills (TASSK-R) and the parent-reported social skills and autism-related difficulties (SRS-2) had large effect sizes, more comparable to the original RCTs of the program and its replications conducted in the U.S. than adaptations delivered in China, South Korea, and Japan (Shum et al., 2019; Yamada et al., 2020; Yoo et al., 2014). Perhaps, Poland is culturally closer to the U.S. than Asian societies and that could have affected both the efficacy and acceptability of the treatment. Alternatively, this may reflect adaptation efforts, such as preparing new role-play videos with Polish actors, instead of adding subtitles to the original videos, as in the previous adaptations. Lastly, the time spent by the first author in training by the original development team was significantly longer than in the case of earlier adaptations which could have improved the author’s understanding of the program.

In the current study, participants’ gains in the SRS-2 were also large compared to other SSTs for young people on the autism spectrum (Wolstencroft et al., 2018). This result was further validated by similar gains in another measure of autism-related difficulties, the ASRS, and specifically in the Peer Socialization subscale.

The number of get-togethers increased significantly in a Treatment Group but decreased in a follow-up by the teen report, with mixed results in the parent report. However, the number of get-togethers has not returned to the initial level and follow-up analyses might not have had sufficient power to detect smaller gains. A similar pattern of results was found in studies delivered in the U.S. (Laugeson et al., 2012; Mandelberg et al., 2014). Perhaps, it reflects the needs or motivational tendencies of autistic teens rather than difficulties in peer relationships due to poor social skills (Sedgewick et al., 2016). Indeed, 2–3 get-togethers with friends per month, as found in the follow-up, may be an adaptive frequency for that population, ensuring regular social contact but protecting from overstimulation. Alternatively, for autistic teens, social engagement is harder to maintain than social skills, which may suggest the need for a maintenance program or booster sessions to promote the former.

The only outcome that showed no change during the intervention was the level of conflict, as reported by teens. Interestingly, parents reported a much higher level of peer conflict before the intervention, while adolescents declared a low level across all data points. These discrepancies may reflect differences in the perception of teens’ relationships or differences in knowledge about them. During the program, parents were encouraged to monitor their teens’ relationships more closely and that could have resulted in a change in their perception.

Overall, the study provided the longest follow-up period among RCTs evaluating the PEERS® program. Maintenance of most of the treatment effects is in line with previous data from secondary analyses showing that gains have not diminished after 1–5 years following the intervention (Mandelberg et al., 2014). However, the poor response rate (58–60%) increased the risk of attrition bias, so the results must be interpreted with caution.

The current adaptation of the PEERS® intervention fills an important gap in evidence-based treatments for adolescents on the autism spectrum in Poland. It also contributes to the still small number of cultural adaptations of social skills training for autistic teens (Davenport et al., 2018), including a few versions of the PEERS® curriculum (Rabin et al., 2018; Shum et al., 2019; Yamada et al., 2020; Yoo et al., 2014). Importantly, it is only second RCT showing the efficacy of PEERS® in Europe, demonstrating the feasibility of such adaptations. Perhaps, as the program is already popular in Europe, some researchers did not see the necessity of conducting its full cultural adaptation and efficacy evaluation. However, some significant changes have been made in the current adaptation regarding language (e.g., meaning and use of a ‘friend’), materials (e.g., preparing new, ecologically valid role-play videos), therapeutic techniques (e.g., discontinuing a token economy), and didactic content (e.g., introducing sending a text message, instead of leaving a voicemail; adjusting jokes, crowds, and teasing comebacks). Therefore, in the light of the study, it is recommended to adapt the program to cultural differences and – even more so – to societal and technological changes that have occurred since the program’s conception. Generational changes in social behavior imply also a necessity for updating existing social skills curriculums. Newer adaptations of PEERS®, including the present one, can inform this process.

The study provides detailed, quantitative and qualitative data on the ecological validity of the intervention. Although the program provided both teens and parents with different experiences than what they had known from typical SSTs in Poland (e.g., doing homework, involvement of parents), the intervention was accepted and deemed helpful. Both sides underscored the concrete, structured, and practical character of the training, making it friendly for autistic teens’ thinking styles. Importantly, the program was not assessed as overly time-burdening, even though homework received relatively the lowest satisfaction scores from adolescents.

By natural experiment, the simultaneous occurrence of the first wave of the COVID-19 and the commencement of this research allowed for the evaluation of the impact of treatment delivery on the therapeutic effects. Results indicate that the hybrid mode of teaching social skills (about half of the classes were held online) did not impact the outcomes negatively. This is in line with emerging evidence that online CBT-based training can be beneficial for autistic people (Conaughton et al., 2017; Hepburn et al., 2016), including a recent quasi-experimental study of the PEERS for Adolescents program via telehealth delivery (Estabillo et al., 2022). Importantly, in the current study, the online environment did not impede key elements of the program, such as behavioral rehearsals, interactions between students, or homework completion. Moreover, giving participants who were unable to participate in the classes in person (due to illness or quarantine) an opportunity to be present online allowed them to maintain high attendance rates (93.75%) throughout the treatment. However, comparing hybrid and in-person delivery was not a planned goal of this study and both interventions took place in different periods, so the results must be treated with caution. Hence, future studies, preferably randomized controlled trials, should include planned, concurrent comparisons of online/hybrid and in-person delivery.

### Limitations

This study is not without limitations. First, it has relied on parent- and self-report with no blinded observers involved, so the results can be subject to detection bias. However, the measure of knowledge about social skills (TASSK-R) was criterion-based and there was general consistency between parent and teen measures of the number of get-togethers (QSQ-R), which is more objective measure of social engagement. Future studies might include behavioral observation measures, such as the Contextual Assessment Social Skills used in some evaluations of PEERS® (Dolan et al., 2016; Idris et al., 2022). Second, using peer and teacher measures could allow for further validation of the generalization of the acquired skills, although parents and teens were asked about the peer context specifically (Peer Socialization subscale of the ASRS, QSQ-R). Third, the outburst of the COVID-19 pandemic forced deviations from the study protocol (i.e. repeating the baseline assessment, hybrid mode of the treatment delivery in TG, prolonged follow-up period) and could limit some of the intervention effects (e.g., the number of get-togethers, due to mobility restrictions). On the other hand, these unforeseen conditions allowed for a preliminary examination of the program efficacy in hybrid mode, which is an additional contribution of this study. However, the comparison of treatment delivery modalities has its own limitations, as the compared interventions took place at different times. Specifically, the Treatment Group experienced more restrictions on mobility and social gatherings than the Waitlist Control Group. Lastly, due to the poor response rate (58–60%) in the follow-up analyses, the maintenance of the treatment effects was not assessed separately in TG and WCG, as well as exposed these findings to attrition bias.

## Conclusion

This study adds to the still limited evidence for the efficacy of the culturally adapted SSTs for adolescents on the autism spectrum. Specifically, it has shown large, durable effects of the PEERS® program on the adolescents’ social skills and knowledge by teen and parent reports, with mixed results regarding the number of get-togethers. Moreover, the study fills an urgent gap in evidence-based SSTs in Poland and proves the feasibility of this kind of parent-mediated CBT intervention. Finally, it shows the efficacy of the hybrid mode of treatment delivery that may be of increasing relevance in the future.

### Electronic supplementary material

Below is the link to the electronic supplementary material.

Supplementary Material 1
