# Supplementary material for: Effects of Social Skills Training for Adolescents on the Autism Spectrum: a Randomized Controlled Trial of the Polish Adaptation of the PEERS® Intervention via Hybrid and In-Person Delivery
Source: J Autism Dev Disord. 2022 Aug 24;53(11):4132–46. doi: 10.1007/s10803-022-05714-9 (PMC9399988; doi:10.1007/s10803-022-05714-9)
Supplement: Supplementary file 1 — Supplementary Material 1 [file 10803_2022_5714_MOESM1_ESM.docx]

Appendix

Table S1. Overview of the Polish adaptation of the PEERS^®^ curriculum

| Session | Didactic lesson | Description | Cultural adaptations and updates |
| --- | --- | --- | --- |
| 1 | Introduction and trading information | Overview of the program.  Characteristics and types of peer relationships.  Rules for trading information. | Following the PEERS for Young Adults curriculum (Laugeson 2017), a discussion on types of peer relationships was added, with some cultural/language differences considered, e.g., a word ‘friend’ is reserved only for very close relationships, a word ‘colleague’ can mean both classmate and someone we like and spend time with.  Simplifying ‘Jeopardy’ game so that it is more similar to regular quiz that participants are acquainted with. |
| 2 | Two-way conversations | Rules for having two-way conversation, including asking open-ended and follow-up questions and non-verbal communication.  Common topics of conversations with peers. | Following the Telehealth version of the PEERS curriculum, new rules were added: Don’t be brutally honest; don’t interrupt. |
| 3 | Electronic communication | Steps for exchanging contact information and for starting and ending a phone call.  Rules for using the Internet.  Rules for using social media. | Leaving a voicemail message is not common among teens in Poland anymore; instead, steps for sending a text message if someone did not answer the phone were introduced.  Types of social media were updated for those popular among Polish teens (Instagram, Facebook, Snapchat, Reddit, TikTok).  Following the Telehealth version of the curriculum, new rules for using the Internet and social media were added.  As it is now common for older teens to turn online friends into real ones, safety rules for such situations were introduced. |
| 4 | Choosing appropriate friends | Brainstorming and identifying appropriate peer groups (crowd) teens belong to or would like to.  Cues for acceptance or rejection in peer relationships. | Modification of the crowds popular among teens: some of the original crowds were not relevant in Poland (e.g., surfers, cheerleaders, preppies) and some new were introduced (e.g., animal lovers, ecological/vegan/vegetarian groups, LGBT+). |
| 5 | Appropriate use of humor | Basic rules for appropriate use of humor.  Paying attention to humor feedback.  Determining one’s own attitude towards joking. | Knock-knock jokes were replaced by jokes used by teens in Poland. |
| 6 | Starting and joining the conversation | Steps for starting individual conversation.  Steps for joining group conversation. | Following the PEERS Curriculum for School-Based Professionals (Laugeson 2013), steps for starting an individual conversation were introduced. |
| 7 | Existing conversation | Steps for exiting the conversation when accepted, initially accepted but then rejected, or rejected. | None. |
| 8 | Good sportsmanship | Rules of good sportsmanship. | None. |
| 9 | Get-togethers | Rules and steps for planning, preparing, starting, and ending a successful get-together, as well as appropriate behaviors when meeting with friends. | Modifications in starting a get-together held at home: a step ‘Show them around’ was omitted as it is not common for Polish teens and a step ‘Take their jacket or coat’ was introduced as it is regularly practiced. |
| 10 | Handling arguments | Rules and steps for responding and bringing up disagreements. | None. |
| 11 | Changing reputations | Rules and steps for changing a bad reputation in a peer group. | None. |
| 12 | Handling teasing and embarrassing feedback | Instructions for appropriate responding to teasing and embarrassing feedback. | Teasing comebacks were adapted so they reflect ecologically valid responses to teasing among Polish teens. |
| 13 | Handling physical bullying | Strategies for dealing with physical bullying (e.g., hitting, forcing to do something, taking things). | Following the PEERS Curriculum for School-Based Professionals, a new rule was introduced: Don’t use teasing comebacks with people that are physically aggressive. |
| 14 | Handling cyber bullying | Strategies for handling bullying on the internet, including social media. | This lesson was added, following the PEERS Curriculum for School-Based Professionals, with no major changes. |
| 15 | Minimizing rumours and gossip | Strategies for handling gossips, including spreading the rumor about oneself. | None. |
| 16 | Graduation | Summary of the program and instructions for maintenance of effects and further development.  Graduation party – social time with boarding games and food and beverages. | A new rule was introduced: ‘Be yourself’ to encourage teens to consider what is important to them and avoid potentially harmful masking strategies. |

Table S2. Teen and parent satisfaction of the PEERS^®^ program

|  | | Treatment Group  (*n* = 12) | | Waitlist Control Group (*n* = 15) | | Summary (*n* = 27) |
| --- | --- | --- | --- | --- | --- | --- |
|  | | *M (SD)* | | *M (SD)* | | *M (SD)* |
| *Teens’ satisfaction* | |  |  |  |  |  |
| How helpful were the following components  of PEERS for you:  (1 - not helpful at all; 7 - very helpful) | |  |  |  |  |  |
|  | Group discussion and role-play demonstrations | 5.5 (1.9) |  | 5.7 (1.0) |  | 5.6 (1.4) |
|  | Behavioral exercises performed  during classes | 5.3 (1.8) |  | 5.9 (1.1) |  | 5.6 (1.4) |
|  | Homework | 4.5 (1.9) |  | 4.4 (1.9) |  | 4.4 (1.9) |
|  | Social coach (parent) support | 5.5 (1.7) |  | 4.8 (1.9) |  | 5.1 (1.9) |
| How do you rate the time burden of participating in the program?  (1 – little burden; 7 – too much burden) | | 2.8 (2.2) |  | 3.8 (2.0) |  | 3.3 (2.1) |
| Do you think that by participating in the program you have learned to establish and maintain friendships better?  (1 – definitely not; 7 – definitely yes) | | 5.8 (1.7) |  | 5.2 (1.5) |  | 5.2 (1.6) |
| Would you recommend this program to other teens on the autism spectrum?  (1 – definitely not; 7 – definitely yes) | | 5.4 (1.8) |  | 6.0 (1.7) |  | 5.7 (1.8) |
| *Parents’ satisfaction* | |  |  |  |  |  |
| How helpful were the following components of PEERS for you:  (scale 1 - not helpful at all; 7 - very helpful) | |  |  |  |  |  |
|  | Homework review during classes | 6.9 (0.3) |  | 6.2 (1.0) |  | 6.5 (0.8) |
|  | Didactic lessons and watching role-play videos during classes | 6.7 (0.8) |  | 6.5 (0.9) |  | 6.6 (0.8) |
|  | Opportunity to watch role-play videos at home | 6.5 (1.2) |  | 6.6 (0.9) |  | 6.6 (1.0) |
| How do you rate your time burden of participating in the program?  (1 – little burden; 7 – too much burden) | | 3.6 (1.3) |  | 3.5 (1.8) |  | 3.6 (1.6) |
| How do you rate your teen’s time burden of participating in the program?  (1 – little burden; 7 – too much burden) | | 3.5 (1.4) |  | 3.7 (1.2) |  | 3.6 (1.3) |
| Do you think that by participating in the program your teen has learned to establish and maintain friendships better?  (1 – definitely not; 7 – definitely yes) | | 5.8 (1.2) |  | 5.9 (1.1) |  | 5.9 (1.1) |
| Would you recommend this program to other parents of teens on the autism spectrum? (1 – definitely not; 7 – definitely yes) | | 7.0 (0.0) |  | 6.9 (0.3) |  | 7.0 (0.2) |
